# Gingipain R1 and Lipopolysaccharide From *Porphyromonas gingivalis* Have Major Effects on Blood Clot Morphology and Mechanics

**DOI:** 10.3389/fimmu.2020.01551

**Published:** 2020-07-24

**Authors:** J. Massimo Nunes, Tristan Fillis, Martin J. Page, Chantelle Venter, Ophélie Lancry, Douglas B. Kell, Ursula Windberger, Etheresia Pretorius

**Affiliations:** ^1^Department of Physiological Sciences, Faculty of Science, Stellenbosch University, Stellenbosch, South Africa; ^2^HORIBA Scientific, HORIBA FRANCE SAS, Villeneuve-d'Ascq, France; ^3^Department of Biochemistry, Faculty of Health and Life Sciences, Institute of Integrative Biology, University of Liverpool, Liverpool, United Kingdom; ^4^The Novo Nordisk Foundation Center for Biosustainability, Technical University of Denmark, Lyngby, Denmark; ^5^Decentralised Biomedical Facilities, Centre for Biomedical Research, Medical University Vienna, Vienna, Austria

**Keywords:** Gingipain R1, lipopolysaccharide, *Porphyromonas gingivalis*, thromboelastography, rheometry, confocal, scanning electron microscopy, Raman

## Abstract

**Background:** *Porphyromonas gingivalis* and its inflammagens are associated with a number of systemic diseases, such as cardiovascular disease and type 2 diabetes (T2DM). The proteases, gingipains, have also recently been identified in the brains of Alzheimer's disease patients and in the blood of Parkinson's disease patients. Bacterial inflammagens, including lipopolysaccharides (LPSs) and various proteases in circulation, may drive systemic inflammation.

**Methods:** Here, we investigate the effects of the bacterial products LPS from *Escherichia coli* and *Porphyromonas gingivalis*, and also the *P. gingivalis* gingipain [recombinant *P. gingivalis* gingipain R1 (RgpA)], on clot architecture and clot formation in whole blood and plasma from healthy individuals, as well as in purified fibrinogen models. Structural analysis of clots was performed using confocal microscopy, scanning electron microscopy, and AFM-Raman imaging. We use thromboelastography® (TEG®) and rheometry to compare the static and dynamic mechanical properties of clots.

**Results:** We found that these inflammagens may interact with fibrin(ogen) and this interaction causes anomalous blood clotting.

**Conclusions:** These techniques, in combination, provide insight into the effects of these bacterial products on cardiovascular health, and particularly clot structure and mechanics.

## Background

Bacterial involvement in inflammatory conditions, via the occurrence of leaky gut (gut dysbiosis) and periodontitis and/or gingivitis, are accompanied by the shedding of cell wall components such as lipopolysaccharides (LPSs) and lipoteichoic acids (LTAs), and these molecules are known to be highly inflammagenic (1–7). The liberation of free iron is often an accompaniment to inflammatory conditions, and we have brought these ideas and data together as the Iron Dysregulation and Dormant Microbes (IDDM) hypothesis of chronic inflammatory and cardiovascular diseases. Many non-communicable diseases have been associated with the presence of periodontitis, gut dysbiosis, bacterial translocation via the gut and increased levels of the bacterial inflammagen, lipopolysaccharide (LPS); for an overview see (5).

Diseases where bacterial involvement has been implicated include Alzheimer's disease (AD) and Parkinson's disease (PD), and disease development and progression has also been linked to periodontitis (8–16). Entrance of bacteria into the body might occur via gut dysbiosis, and impaired gut health is also present in both AD (15, 17) and PD (18–21). Type diabetes (T2DM) is also associated with bacterial translocation via the gut (22–24). LPS presence has also been noted in the central nervous system of patients with AD (25–27) and in their blood (28). LPS has also been implicated in T2DM (29, 30), sepsis (31), rheumatoid arthritis (32), and psoriasis vulgaris (33).

*Porphyromonas gingivalis* is a well-known bacterium that causes periodontitis and gingivitis (34, 35), and its inflammagens have been associated with the development of various inflammatory conditions (36–40). *P. gingivalis* and its inflammagens are associated with cardiovascular disease and T2DM (41). Except for the presence of its cell wall inflammagen LPS, *P. gingivalis* also produces an unique class of cysteine proteinases termed gingipains. Live *P. gingivalis*, as well as its LPS, are powerful peripheral and intracerebral inflammatory signaling initiators (42). LPS from *P. gingivalis* also acts via the Toll-like Receptor (TLR) signaling pathway the authors studied the TLR4 signaling pathway in C57BL/6 mice (43). Recently, Dominy and co-workers provided clear evidence that *P. gingivalis*, and more specifically, it protease, gingipains, play a fundamental role in the development of AD (40). They discovered gingipains in the brain lesions of AD patients (40). Gingipains consist of Arg-gingipain (Rgp) (RgpA and RgpB), and Lys-gingipain (Kgp), and play a central role in the virulence of this organism (44). Gingipains cleave proteins toward the C-terminal after arginine or lysine residues and are classified accordingly: gingipain R is arginine-specific and gingipain K is lysine-specific. This proteolytic activity of gingipains play a crucial role in the physiology of the bacterium, where it is essential for obtaining nutrients via protein degradation, for adherence to host surfaces and for further colonization (45). Gingipains are also known to exert fibrin(ogen)olytic activity and when present in circulation, can interact and cleave plasma proteins (46–48).

By definition, inflammation is normally accompanied by the production of inflammatory cytokines, such as interleukins (ILs) IL1β, IL6, and TNF-α, some bacterial inflammagens such as LPS are well-characterized, and more recently implicated in inflammatory conditions, but overall, little is known on how bacterial inflammagens act as biomarkers in the various inflammatory conditions (2, 4, 5). Inflammation is also an almost inevitable accompaniment of cardiovascular disease, but a much less recognized feature of inflammation is coagulopathies (49–51). We recently discovered that, in part, these coagulopathies were represented by the clotting of blood into an anomalous form, and that this can be catalyzed by miniscule amounts of LPS (from *E. coli*) or LTA (10^−8^ mol/mol fibrinogen) (2, 52). Recently, it was also noted that LPS from *P. gingivalis* added to platelets cause significant morphological changes to platelets (53). Platelets exposed to this LPS showed spreading, with increased presence of actin-rich filopodia, by activation of Cdc42, the small GTPase responsible for filopodia formation. Exposure of whole blood samples to LPS from *P. gingivalis* also significantly reduced clotting times (53).

Because of the findings of Dominy et al. where they detected gingipains in AD brain lesions (40) and our interest on how bacterial inflammagens interact with clotting proteins, we searched for the presence of gingipains in the serum of patients with PD (54). We detected RgpA from *P. gingivalis* in PD plasma using fluorescent antibodies and found significantly increased levels of this protease compared to age-matched controls.

Because there are numerous reports that *P.gingivalis* and its inflammagens are important contributary agents in neuroinflammatory, as well as cardiovascular conditions, including T2DM, the question now arose as to how gingipains and LPS from *P. gingivalis* interact with circulating plasma proteins. Microbial translocation from inflamed periodontal pockets into coronary atheroma via systemic circulation is also one of the proposed pathways that links periodontitis and myocardial infarction (55). We therefore seek to get specific answers with regards to their effects on both morphology and mechanics of clots. Therefore, in the present study, we investigate the effects of the bacterial products LPS from *E. coli* and *P. gingivalis*, as well as the gingipain RgpA *P. gingivalis* [recombinant *P. gingivalis* Gingipain R1 (RgpA)], on clot architecture and clot formation in whole blood and plasma from healthy individuals, as well as in purified fibrinogen models. Structural analysis of clots was performed using confocal microscopy, scanning electron microscopy and AFM-Raman imaging. We use thromboelastography® (TEG®) and rheometry to compare the static and dynamic mechanical properties of clots.

To investigate our hypothesis, the various analyses were done in various laboratories. We therefore included a large variety of equipment and sample preparation methods and used optimized and well-established protocols from each laboratory. These various techniques in combination provide insight into the effects of these bacterial products on coagulation, and particularly clot structure and mechanics. We found that these inflammagens may interact with fibrin(ogen) and cause blood to clot abnormally (anomalous clotting). These results are in line with our previous findings of LPS from *E. coli*, and we further show here that LPS from *E. coli* influences the clot structure of purified fibrin(ogen) (2). Furthermore, understanding how bacterial inflammagens interact with plasma proteins, when in circulation, may result in a better understanding of clot and coagulation pathologies in inflammatory conditions. Ultimately, we may find solutions to treat pathological clotting, driven by bacterial inflammagens, as pathological clotting is an important co-morbidity to most inflammatory conditions.

## Materials and Methods

### Study Design and Ethical Statement

The present study uses a cross-sectional study design. Ethical clearance was obtained from the Health Research Ethics Committee (HREC) of Stellenbosch University, South Africa (N19/03/043) and from the Ethics Committee of the Medical University Vienna, Austria (EK1371/2015). Written informed consent was obtained from all participants followed by whole blood sampling. Study participants received a unique number that was used to guarantee anonymity throughout this study, and researchers followed Good Clinical Practice and guidelines from the ethics committee.

### LPS and Gingipain (RgpA)

The bacterial analytes that were added to plasma and fibrinogen were prepared in endotoxin-free water and they are:

- RgpA (Abcam. ab225982); purity is at >90% SDS-PAGE
- *E. coli* LPS (Sigma, L2630) and *P. gingivalis* LPS (Sigma SMB00610). Both the LPSs' purity is MQ300, which is stipulated for products used in applications requiring enhanced change control and quality agreement. However, it is noted that Jain et al. (56) reported that some LPS preparations might have lipoprotein contaminants present.

### Purified Fibrin(Ogen) Clot Model

We used three purified fibrin(ogen) clot models: (1) fluorescent fibrinogen conjugated to Alexa Fluor™488 (ThermoFisher, F13191), (2) non-conjugated purified fibrinogen (Sigma, F3879) and (3) non-conjugated purified fibrinogen depleted of von Willebrand factor, plasminogen, and fibronectin (CoaChrom, HFG3). These products were also prepared in endotoxin-free water.

### Participants and Blood Collection

Healthy volunteers [*N* = 39; 23 females, 16 males; median age (interquartile range): 42] were recruited for this study. The inclusion criteria for healthy volunteers were: non-smokers, absence of infection, no use of anti-inflammatory or chronic medication, and no previous history of thrombotic disease, neurological diseases like AD and PD, or T2DM. An exclusion criterion was the presence of both gingivitis and periodontitis. Blood was drawn in serum-separating, EDTA, and sodium citrate tubes by a phlebotomist. After the blood was drawn, whole blood samples were allowed to rest for 30 min at room temperature before further processing for experimentation. Two plasma derivatives were created. Platelet poor plasma (PPP) was created by centrifuging whole blood at 3,000 g for 15 min. The plasma fraction was collected and stored at −80°C until experimentation. For rheometry analysis, platelet-depleted plasma (PDP) was created by centrifuging whole blood at 325 g for 8 min. After removal of the surface layer, the top two-thirds of this plasma were collected and centrifuged a second time at 2,310 g for 30 min, and the top two-thirds ultimately used for experimentation.

### Scanning Electron Microscopy

#### Platelet Poor Plasma With LPS

A scanning electron microscope was used to view the ultrastructural changes of clots. PPP was exposed to LPS from *P. gingivalis* (*n* = 10; 10 ng.L^−1^; 30 min) before creating a plasma clot on a 10 mm glass cover slip with the addition of thrombin (7 U·mL^−1^). South African National Blood Service. Matching naïve clots were prepared with addition of thrombin. The samples were then washed in PBS followed by a fixation step of 4% formaldehyde and secondary fixation in 1% osmium tetroxide (OsO_4_), with PBS wash steps in between. This was followed by serial dehydration in ethanol and hexamethyldisilazane (HMDS). The samples were coated with carbon and viewed with a Zeiss MERLIN FE-SEM with the InLens detector at 1 kV. All SEM images (3 images per clot) were analyzed using ImageJ where fibrin fiber width was assessed for each image using a grid overlay to accurately record these measurements. The central fibers in 12 squares on each image were measured.

#### Purified Fibrinogen With LPS and RgpA

Purified fibrinogen (CoaChrom) was incubated with the following substances and prepared in technical triplicate following the above protocol, with the exception of using 0.15 U·mL^−1^ alpha-thrombin (CoaChrom, HF2A) to create the clots. Samples were viewed as above.

- RgpA at 20 μg·L^−1^
- LPS from *P. gingivalis* at 5, 20, and 20 μg·L^−1^
- LPS from *E. coli* O111:B4 at 5, 20, and 20 μg·L^−1^
- Combination of RgpA (20 μg·L^−1^) and LPS *P. gingivalis* (20 μg·L^−1^)

### Confocal Microscopy on PPP With LPS

Platelet poor plasma was exposed to LPS from *P. gingivalis* (*n* = 10; 10 ng·L^−1^; 30 min) and clotted with thrombin (7 U·mL^−1^) on a microscope slide to create a fibrin fiber clot. Exposed clots were compared to their matched naïve samples by visualizing intrinsic fluorescence on a Zeiss LSM 780 confocal microscope with a Plan-Apochromat 63x/1.4 oil DIC M27 objective. Clotted samples were excited by the 488 nm laser with emission detected between 508 and 570 nm and by the 561 nm laser with emission detected between 593 and 700 nm. These settings were chosen after scanning the samples with the hyperspectral mode of the confocal with each laser and determining the best emission range for autofluorescent signal in these samples. The area coverage of the autofluorescent signal in the confocal images was analyzed using ImageJ, with differences in the autofluorescent signal taken to reflect differences in the structure of the clot. Thresholding between 26 and 255 on the greyscale provided a consistent analysis of the images (3 images per clot). The percentage fluorescent area to total area of each image was compared between control and LPS-exposed groups.

### Confocal Microscopy With Airyscan on Fluorescent Fibrinogen With LPS

Fluorescently labeled Alexa Fluor™ 488 purified fibrinogen (2 mg·mL^−1^) was used to evaluate anomalous clotting, upon the addition of *P. gingivalis* LPS to the fibrinogen (100 ng·L^−1^; 30 min). Samples were clotted with thrombin (7 U·mL^−1^) on a microscope slide and viewed with the Zeiss MP880 confocal microscope in Airyscan mode. Exposed clots were compared to their matched naïve samples by exciting the fibrin fibers with the 488 nm laser and collecting the emission with band pass filters 420–480 and 495–550 nm.

### Confocal Microscopy on Fluorescent Fibrinogen With LPS

Fluorescently labeled Alexa Fluor™ 488 purified fibrinogen (2 mg·mL^−1^) was exposed to *E. coli* LPS (20 ng·L^−1^; 30 min) or *P. gingivalis* LPS (20 ng·L^−1^; 30 min). Naïve and LPS-exposed samples were clotted with thrombin (7 U·mL^−1^) on a microscope slide and viewed on a Zeiss LSM 780 confocal microscope with a Plan-Apochromat 63x/1.4 oil DIC M27 objective. Images were captured in lambda mode with the 488 nm laser and the GaAsP detector, which measures fluorescent emission between 410 and 695 nm across 32-channels, at 8.9 nm intervals. Multidimensional images were acquired as z-stacks and processed as maximum intensity projections in the ZEN software.

### Correlative Atomic Force Microscopy and Raman Microspectroscopy on Fibrinogen With LPS

AFM-Raman was used to analyse the potential fiber structure changes in purified fibrinogen upon exposure to LPS from *P. gingivalis* (100 ng·L^−1^; 30 min). Purified fibrinogen was clotted on 10 mm gold-coated coverslips (HORIBA Scientific, France) with thrombin (7 U·mL^−1^). Naïve clots were prepared with the addition of thrombin. The glass coverslips were allowed to dry for about 2 min, before being submerged in PBS, followed by fixation in 4% formaldehyde and 1% osmium tetroxide, with PBS wash steps in between. Samples were dehydrated in increasing grades ethanol, before an ultimate HMDS drying step.

The characterization of the samples was performed with a LabRAM Nano. This multi-analysis platform consists of a Raman microspectrometer (LabRAM HR Evolution, HORIBA) combined with an AFM (SmartSPM, HORIBA Scientific) for chemical and physical analysis of the same samples area. The system is based on a reflection configuration capable of approaching the objective lens (Mitutoyo, 100× magnification, NA = 0.7, 20 mm working distance) from top illumination to the sample surface. Incident light is focused through the objective lens onto the apex of the AFM tip probe. In this study, micro-Raman images were measured with the 473 nm laser as the excitation source (3 mW maximum at the sample). Initially, three different wavelengths (473, 532, and 633 nm) were tested. It was determined that the 473 nm was the best choice, and the Raman spectrum was measured in one window. The LabRAM Nano is equipped with an Edge filter to cut the Rayleigh signal so that the Stokes signal could be measured. Raman images were collected from 10 μm^2^ regions with 0.3 μm pixel steps. Acquisition time of each Raman spectrum is 30 s (one spectrum/image pixel). Correlated AFM images were obtained in AC mode using an ACCESS-NC Silicon probe (*k* = 25–95 N/m, *f* = 200–400 kHz, AppNano, US). The shape of the probes allows a direct visualization of the tip apex, which permits correlation with the excitation Raman. AFM images were acquired from 20 × 10 μm areas (300 × 150 pts) for the control sample and 20 × 20 μm areas (300 × 300 pts) for the experimental sample.

### Viscoelastic Analysis

The Thrombelastograph® (TEG®) 5000 Hemostasis Analyzer (Haemoscope Corp) was used to measure the viscoelastic properties of blood, with the measured parameters listed in Table 1. PPP samples were exposed to LPS from *P. gingivalis* (*n* = 10; 10 ng·L^−1^) or RgpA (*n* = 30; 500 ng·L^−1^) for 30 min, with exposed samples compared to their matched naïve samples. (Initially we also exposed samples for 1 h, however, a longer exposure time did not significantly change the TEG® results). Prepared PPP was placed in a TEG® cup, together with 0.01 M calcium chloride (CaCl_2_) to activate the coagulation process. The process was allowed to run until maximal amplitude (MA) was reached.

**Table 1:** TEG® parameters [modified from (57)].

**Thromboplastic parameters**	**Description**
R: Reaction time (minutes)	Time of latency from start of test to initial fibrin formation (amplitude of 2 mm); i.e., initiation time
α angle: (slope between the traces represented by R-time at 2 mm and K-time at 20 mm) (degrees)	The angle measures the speed at which fibrin build up and cross linking takes place, hence assesses the rate of clot formation; i.e., thrombin burst
MA: Maximal amplitude (mm)	Maximum strength/stiffness of clot. Reflects the ultimate strength of the fibrin clot; i.e., overall stability of the clot
MRTG: Maximum rate of thrombus generation (Dyn·cm^−2^·s^−1^)	The maximum velocity of clot growth observed or maximum rate of thrombus generation using G, where G is the elastic modulus strength of the thrombus in dynes·cm^−2^
TMRTG: Time to maximum rate of thrombus generation (minutes)	The time interval observed before the maximum speed of the clot growth
TTG: Total thrombus generation (Dyn·cm^−2^)	The clot strength: the amount of total resistance (to movement of the cup and pin) generated during clot formation. This is the total area under the velocity curve during clot growth, representing the amount of clot strength generated during clot growth

### Rheometry of WB and PDP With LPS and RgpA

Whole blood (WB) (*n* = 2) and PDP (*n* = 2) were subjected to rheometry analysis on a Physica MCR 301 rheometer (Anton Paar, Austria) equipped with a Peltier controlled stainless steel sand-blasted cone-plate system (diameter 50 mm), mounted by a tempered hood and an evaporation blocker filled with silicon oil. The Rheocompass™ software (v1.22, Anton Paar, Austria) was used for data acquisition.

Samples were prepared by exposing blood from control donors for 1 h to either (1) LPS from *E. coli* (20 ng·L^−1^), (2) LPS from *P. gingivalis* (20 or 20 μg·L^−1^), or (3) RgpA (100 or 250 ng·L^−1^). Matching control runs were diluted with the same volume of vehicle as for the exposed samples. Experiments were run in technical triplicate.

Whole blood and plasma were clotted by addition of 0.01 M CaCl_2_ and clots were generated in the cone-plate geometry. A constant sinusoidal strain amplitude (0.1%, 1.5 Hz) was set to observe the process of clot formation with minimal interference. These time sweeps were conducted until a G' plateau was reached, at which point an amplitude sweep test was started. The amplitude sweep tests were stress-controlled with a logarithmic ramp from 1 to 5,000 Pa at constant angular frequency (ω = 1 rad s^−1^).

**Figure 1:**
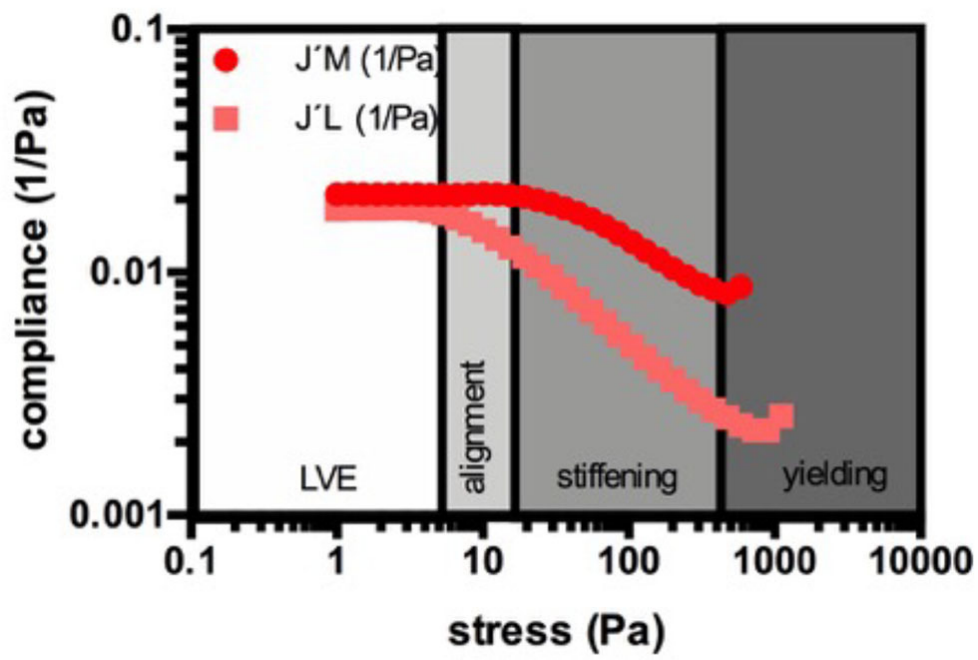
Example for the suggested regions during the strain response of a naïve PDP clot. The minimum-strain compliance shown here (J'_M_) reflects the tangent modulus at zero instantaneous stress, whereas the large-strain compliance (J'_L_) reflects the secant modulus at maximum stress. During the linear viscoelastic behavior (LVE) the clot is in its equilibrium and the compliances merge and remain constant. With increasing stress the compliances start to diverge, which marks the elastic limit and the onset of the non-linear behavior. Now, the network elongates depending on its individual architecture. This period is characterized by progressive alignment of singular structures in the clot to establish a new architecture in which all structures are stretched out (here the microscopical changes in the clot take place). As soon as both compliances decrease together, the phase of shear-stiffening starts. The stretched network elongates as a whole and stiffens when the loads increase. When the stress becomes too high, the clot breaks either abruptly or yields. Yielding includes breaking of branch points or singular fibers in the network.

The rheometry parameters discussed in this paper are given in Table 2. During the amplitude sweep tests, we continuously monitored the resulting strain (γ(ω)) of the material, which is the response of the clot to the applied sinusoidal stress (τ(ω)). The shift of the phase angle (δ) allows the calculation of the storage modulus (G') by multiplying the stress-strain relationship (τ(ω)/γ(ω)) with cos(δ). G' serves as a measure of the reversibly stored and thus recoverable deformation energy and represents clot stiffness. As long as G' is maintained while the shear stress increases, the clot remains in its linear viscoelastic range (in its equilibrium) and experiences only elastic deformation. The clot can return into its initial form when the sinusoidal stress input crosses the 0-point. This can be also seen in the output waveform signal, which remains sinusoidal. With the continuous increase of shear force, a deviation from the initial G' value and a change in the output waveform signal occurs, which marks the onset of the non-linear response. From this shear stress onwards, the clot cannot return into its initial equilibrium state since the stronger deformation does not allow full recovery. The borderline between the linear and the non-linear behavior marks the elastic limit of the clot. As stresses become higher, non-linearity increases until the clot breaks. Since G' can be a misleading measure of the elastic modulus of plastically deforming clots, because other harmonic components may also store energy [see (58)], we applied the model of Ewoldt et al. (59), which is integrated in the Rheocompass software, to calculate clot compliance out of Bowditch-Lissajous plots using an approach that is geometrically motivated (60). The minimum-strain compliance shown here (J'_M_) reflects the tangent modulus at zero instantaneous stress, whereas the large-strain compliance (J'_L_) reflects the secant modulus at maximum stress (Figure 1). At equilibrium (linear clot behavior), both compliances merge, whereas out of equilibrium they diverge (non-linear behavior). Certain points on the curves indicate certain processes in the network, e.g., fiber bending and stretching out network inhomogeneities at intermediate shear stresses, stretching of the clot as a whole in shear direction at higher shear stresses, and weakening or even breaking of network points prior to complete breakup at highest-most shear stresses. Figure 1 shows these suggested regions. We propose that not only an upshift or downshift of the curves—indicating higher or lower compliances—must be considered to classify clots, but also changes in the shape of the compliance curves as they indicate specific clot behaviors. For example, the stress needed to fully stretch out the clot as a whole indicates the end of microscopic processes within the fiber network. Only if all branch points and inhomogeneities are aligned to the force lines, the clot stretches as a whole, which is referred to as macroscopic shear stiffening.

**Table 2:** Rheometry parameters.

**Rheometry parameter**	**Description**
G'_LVE (linear viscoelastic range)_	Stability of the clot at rest—this means at equilibrium conditions. Elastic behavior (reversible deformation) of the clot
Elastic limit	Start of non-linear deformation. The clot cannot relax into its original state beyond this critical shear stress
Breakup stress	Shear stress needed to either break the clot apart or break it from the rheometer plate to which it adheres

### Statistical Analysis

Statistical analyses were performed on GraphPad Prism 7.04 with values of significance stated at *p* < 0.05. All data were subjected to Shapiro-Wilks normality tests. A paired *T*-test was performed on parametric data with the data expressed as mean ± standard deviation, whereas the Mann-Whitney *U*-test was used on unpaired non-parametric data and the Wilcoxon matched-pairs signed rank test was used on non-parametric data that was paired with the data expressed as median [Q1–Q3] (all two-tailed).

## Results

### Scanning Electron Microscopy of PPP Clots With LPS

**Figure 2:**
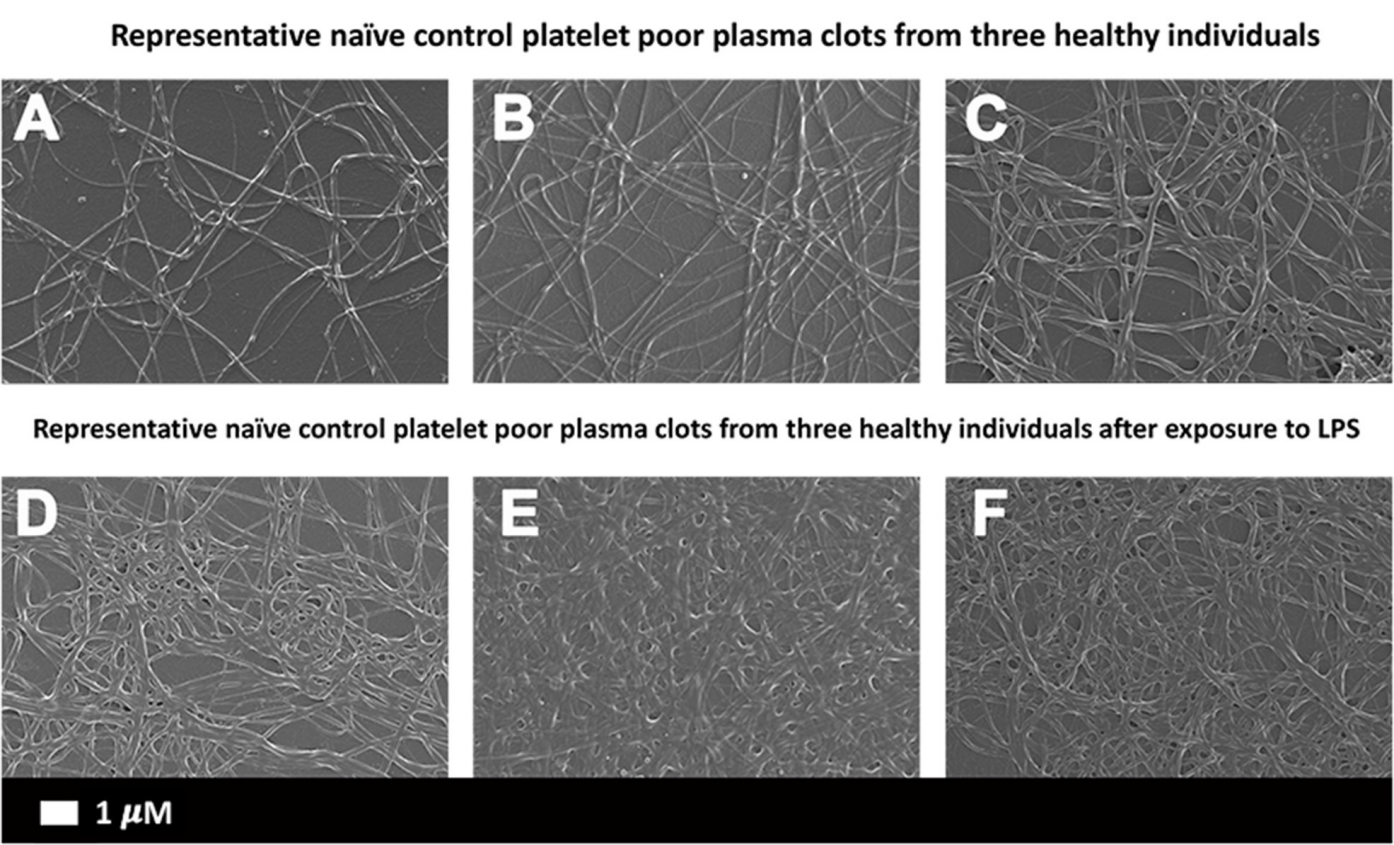
Scanning electron micrographs of **(A–C)** representative naïve control plasma clots and **(D–F)** matched clots with added LPS from *P. gingivalis* (Scale bar: 1 μm).

The differences in PPP clot ultrastructure in naïve clots (Figures 2A–C) and in the presence of *P. gingivalis* LPS (Figures 2D–F) were evaluated. Statistical analysis of fibrin fiber thickness showed a significant (*p* < 0.0001) increase in fiber width between naïve [0.19 μm (0.14–0.25)] and LPS-exposed [0.27 μm (0.2–0.37)] samples.

### Scanning Electron Microscopy of Purified Fibrinogen Clots With LPS and RgpA

**Figure 3:**
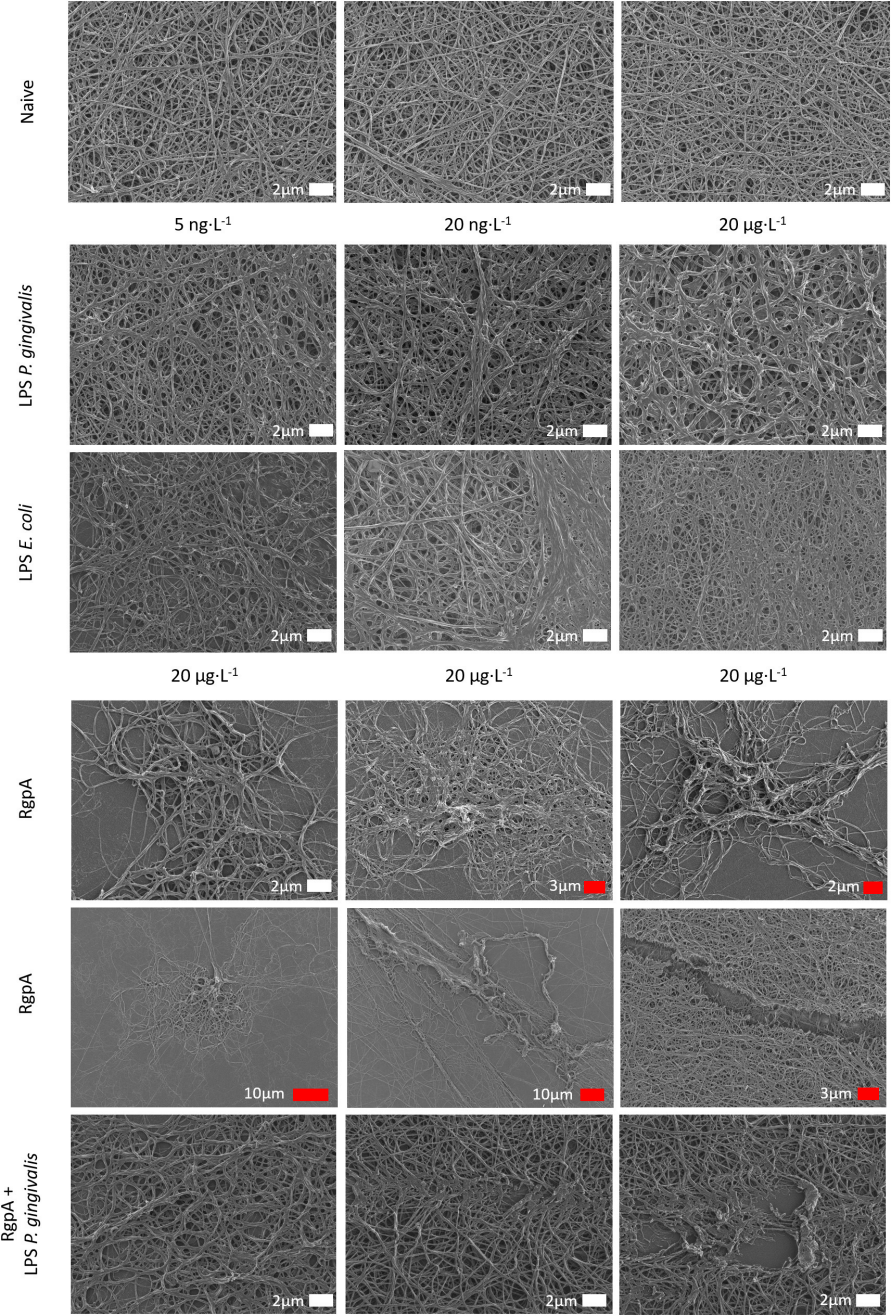
Scanning electron micrographs of purified fibrinogen clots exposed to bacterial products at varying concentrations (white scale bars indicate identical scales).

The effect of LPS from *P. gingivalis* and *E. coli* as well as RgpA on the network structure of pure fibrin fiber networks was examined by SEM (Figure 3). Addition of either LPS from *E. coli* or *P. gingivalis* resulted in greater observations of fused and thicker fibers. Fiber width was statistically greater (*p* < 0.0001) in clots exposed to LPS from *P. gingivalis* [0.22 μm (0.17–0.33)] compared to naïve clots [0.18 μm (0.13–0.22)]. Previously, we reported the same changes in fiber thickness for LPS form *E. coli* (2). Exposure to RgpA led to various changes in the network architecture of fibrin clots. Most fibers were observed as looser networks of clumped fibers, sporadically distributed throughout the SEM preparation and with disruptions to the fiber structure. In the few areas of confluent fibers, some breaks and disruptions to the network could be noted. The combination of RgpA and LPS from *P. gingivalis* seemed to cause breaks and disruptions in the regular fiber networks.

### Confocal Microscopy of PPP Clots With LPS

**Figure 4:**
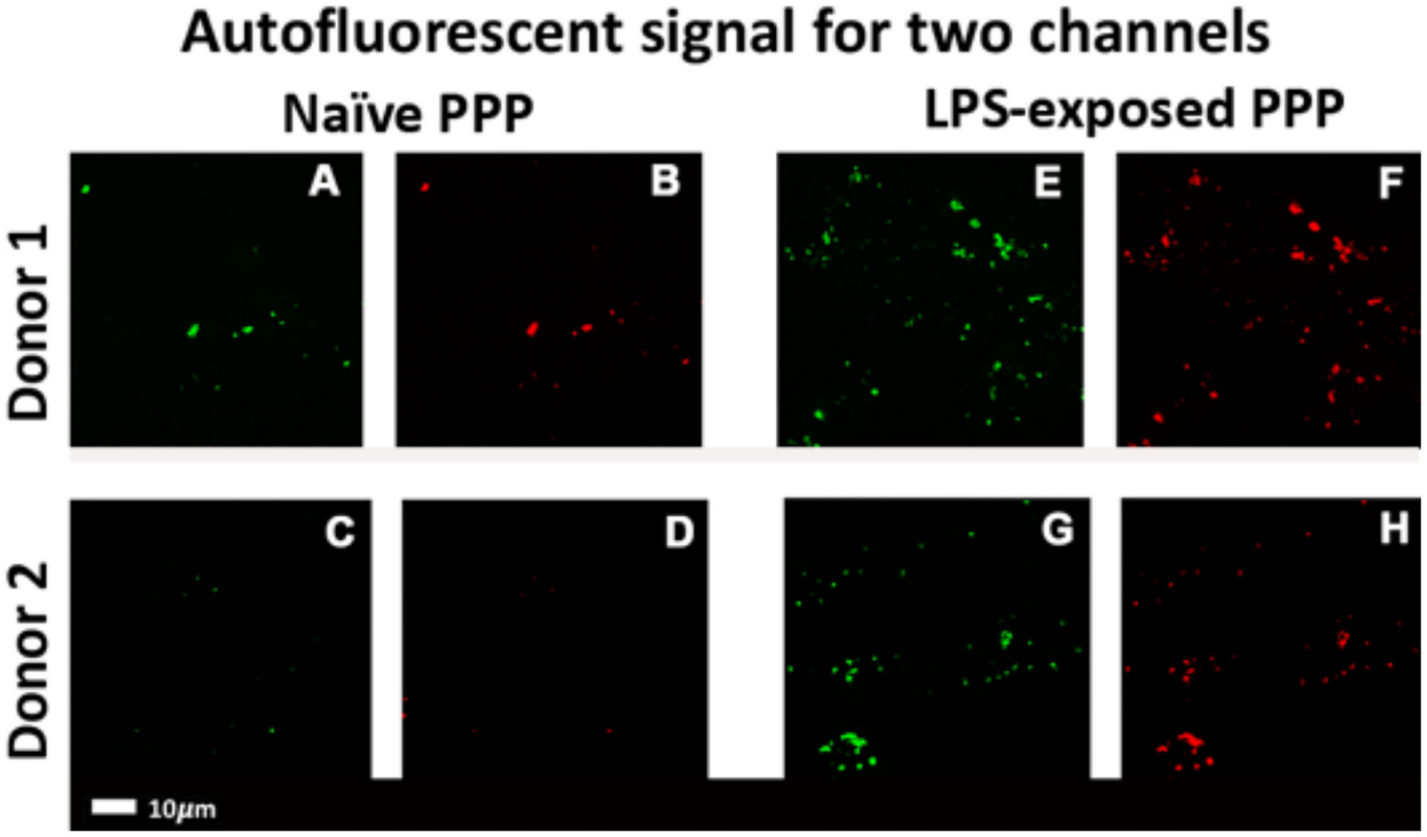
Confocal micrographs of representative clots prepared from PPP of healthy individuals that were exposed to LPS from *P. gingivalis*. Autofluorescence signals were captured in two channels. Green fluorescence: 488 nm laser with a 508–570 nm detector; red fluorescence: 561 nm laser with 593–700 nm detector. **(A–D)** Representative naïve control clots. **(E–H)** Clots with added LPS from matched controls (Scale bar: 10 μm).

Figure 4 shows representative micrographs of the autofluorescence signal in control and LPS-exposed clots for the 488 and 561 nm lasers. The total autofluorescent area of the clots after LPS exposure [1.01% (0.8–1.4)] was significantly (*p* < 0.001) increased compare to the control [0.16% (0.067–0.31)]. Changes in the intrinsic optical properties of fibrinogen might reflect changes to fibri(ogen) (61).

### Confocal Microscopy on Fluorescent Fibrin(Ogen) Clots With LPS

**Figure 5:**
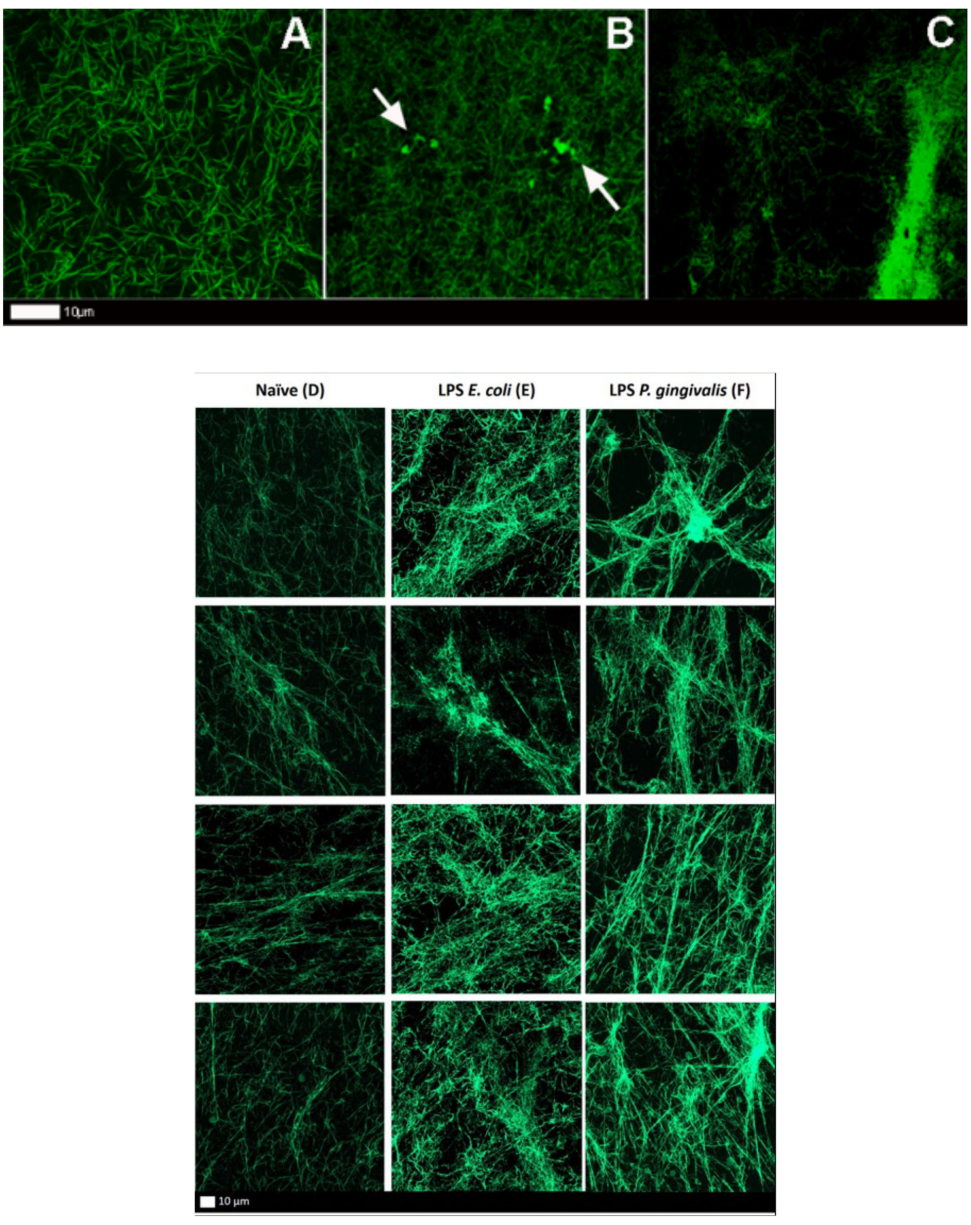
**(A–C)** Airyscan micrographs of fluorescent fibrinogen. **(A)** Naïve clot showing a normal distribution of fibrin fibers. **(B,C)** LPS-exposed (*P. gingivalis*) fluorescent fibrinogen, where plaque-type areas are present (white arrows) (Scale bar: 10 μm). **(D–F)** Confocal lambda maximal intensity projections of fluorescent fibrinogen. Each column shows four representative projections per exposure. **(D)** Naïve clot. **(E)** LPS-exposed (*E. coli*) clot. **(F)** LPS-exposed (*P. gingivalis*) clot.

We also investigated protein misfolding in fluorescent fibrinogen using Airyscan technology (Zeiss MP880), after addition of LPS from *P. gingivalis*. The control fibrin(ogen) clot (Figure 5A) showed typical netted fibrin fibers, whereas the LPS-exposed samples (Figures 5B,C) show areas of intense fluorescence and have a more densely formed fibrin network. Additionally, confocal z-stacks (Zeiss LSM 780) of fibrinogen exposed to *E. coli* and *P. gingivalis* also illustrated changes in the fibrinogen network structure. The control clots (Figure 5D) showed loose networks of fibers, whereas LPS-exposure (Figures 5E,F) show much denser fibers networks, a feature of hypercoagulation.

### Correlative Atomic Force Microscopy and Raman Microspectroscopy on Fibrinogen With LPS

**Figure 6:**
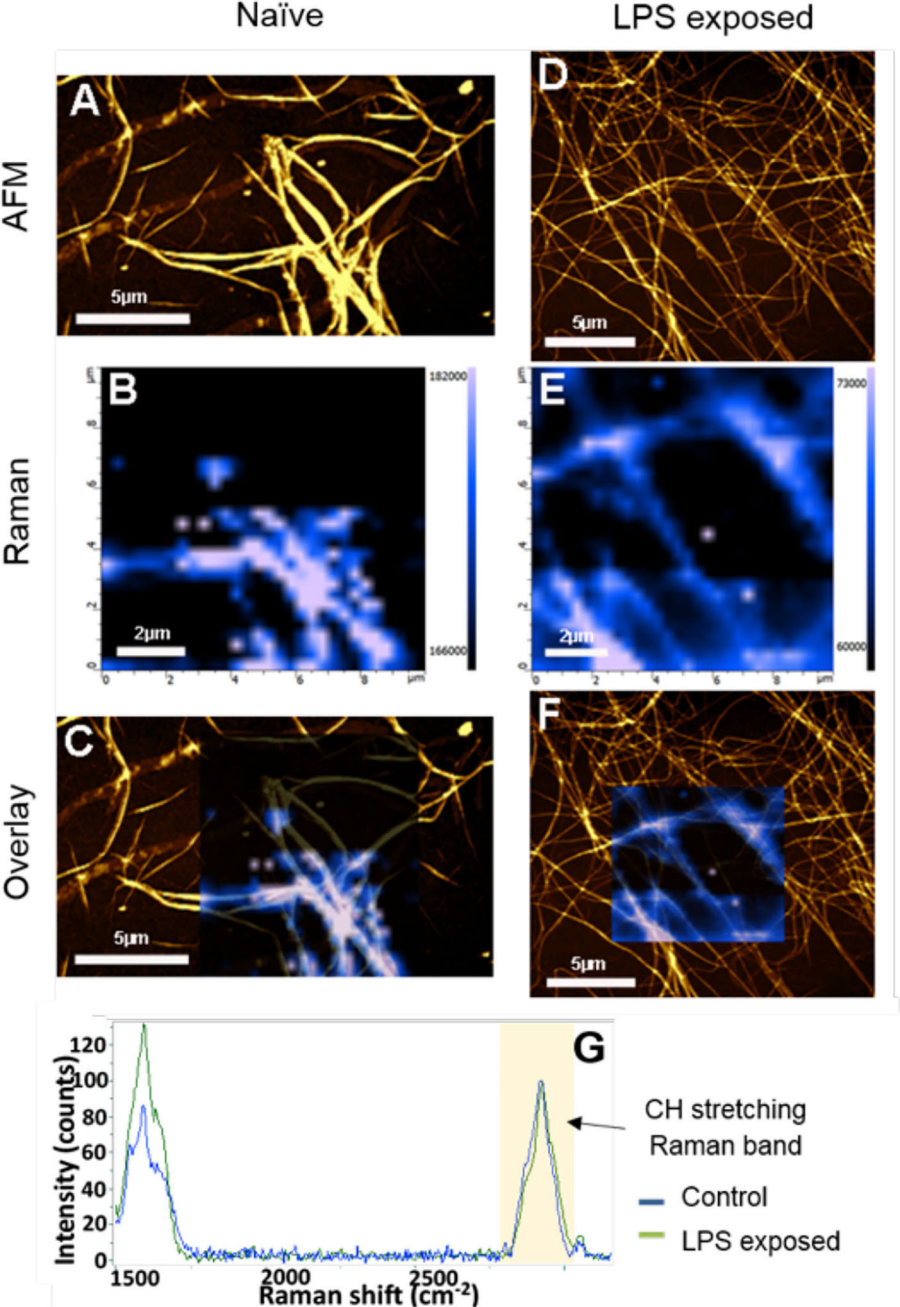
AFM, Raman, and AFM-Raman correlative images of the **(A–C)** control and **(D–F)** *P. gingivalis* LPS-exposed fibrinogen, as well as **(G)** the average Raman spectra of control sample (blue Raman spectrum) and LPS-exposed sample spectra (green Raman spectrum) The Raman images are the Amide I intensity maps of naïve clots vs. LPS-exposed clots. They are overlaid to the AFM images **(B,D)**. Blue spectrum is average spectrum of a Raman map of the naïve sample. Green spectrum is average spectrum of a Raman map of LPS-exposed sample [Scale bars: **(A,C,D,F)**: 5 μm; **(B,E)**: 2 μm].

Correlative AFM and Raman images were obtained from naïve and LPS-exposed samples (Figure 6). The amide I intensity Raman band monitoring (Figures 6B,D) showed a higher Raman signal intensity on the fibers and perfect correlation with the AFM topography. The comparison between the average spectra from the two samples (after C-H stretching band intensity normalization) highlights some slight differences as band broadening, band position shift, and intensity ratio changes, which could indicate a possible ß-sheet unfolding in the LPS-exposed samples (Figure 6E).

### Thromboelastography® of PPP With LPS and RgpA

Table 3 shows the TEG® results for PPP exposed to LPS from *P. gingivalis*, compared to matched naïve samples. Significant changes are seen in the *R*-value, α angle and TMRTG values, indicating accelerated clot formation and fiber cross-linking. This suggests that LPS-exposed clots forms faster, which is a feature of hypercoagulability.

**Table 3:** TEG® results of naïve and *P. gingivalis* LPS-exposed control PPP.

**Parameter**	**Control**	**LPS**	***p*-value**
R	13.80 ± 2.73	10.04 ± 2.73	0.02 (^*^)
α angle	55.61 ± 5.33	60.28 ± 3.16	0.04 (^*^)
MA	22.75 ± 3.36	21.05 ± 4.94	0.3
MRTG	2.73 ± 0.84	3.28 ± 0.76	0.09
TMRTG	15.74 ± 3.26	10.77 ± 2.16	0.007 (^**^)
TTG	148.61 ± 28.36	135.22 ± 40.48	0.3

Data are represented as mean ± standard deviation. Statistical significance was established at p < 0.05 (

* *p < 0.05*;

** *p < 0.01)*.

The effect exerted by the RgpA protease on viscoelastic parameters of clotting is shown in Table 4. All six parameters assessed exhibited significant changes. RgpA pre-treatment shifts the coagulability to a more hypocoaguable state in terms of clotting time, which is represented by the three time-dependent parameters *R*-value (^**^), MRTG (^***^), and TMRTG (^**^), which are all increased compared to controls. The lower α-angle (^**^) reflects that the fibrin build-up is slower in the exposed samples, resulting in a reduction of fibrin cross-linking. In addition, the resultant clot strength and stability measured by MA (^*^) was increased in the RgpA group, whereas strength measured by TTG (^*^) was decreased.

**Table 4:** TEG® results of naïve control and RgpA-exposed PPP.

**Parameter**	**Control**	**RgpA**	***p*-value**
R	9.15 [7.8–11.8]	11.5 [8.13–13.53]	0.0011 (^**^)
α angle	66 [59.73–68.95]	59.63 ± 8.89	0.0014 (^**^)
MA	24.57 ± 6.21	23.45 [18.5–25.15]	0.021 (^*^)
MRTG	4.2 ± 1.72	3.07 [2.17–4.56]	0.0001 (^***^)
TMRTG	10.42 [8.9–13.38]	13.75 [8.96–17.19]	0.0032 (^**^)
TTG	167.7 ± 56.79	149.8 ± 43.28	0.022 (^*^)

Data are represented as either mean ± standard deviation or median [Q1–Q3]. Statistical significance was established at p < 0.05 (

* *p < 0.05*;

** *p < 0.01*;

*** *p < 0.001)*.

### Rheometry of Whole Blood (WB) and Platelet Depleted Plasma (PDP) With LPS and RgpA

**Figure 7:**
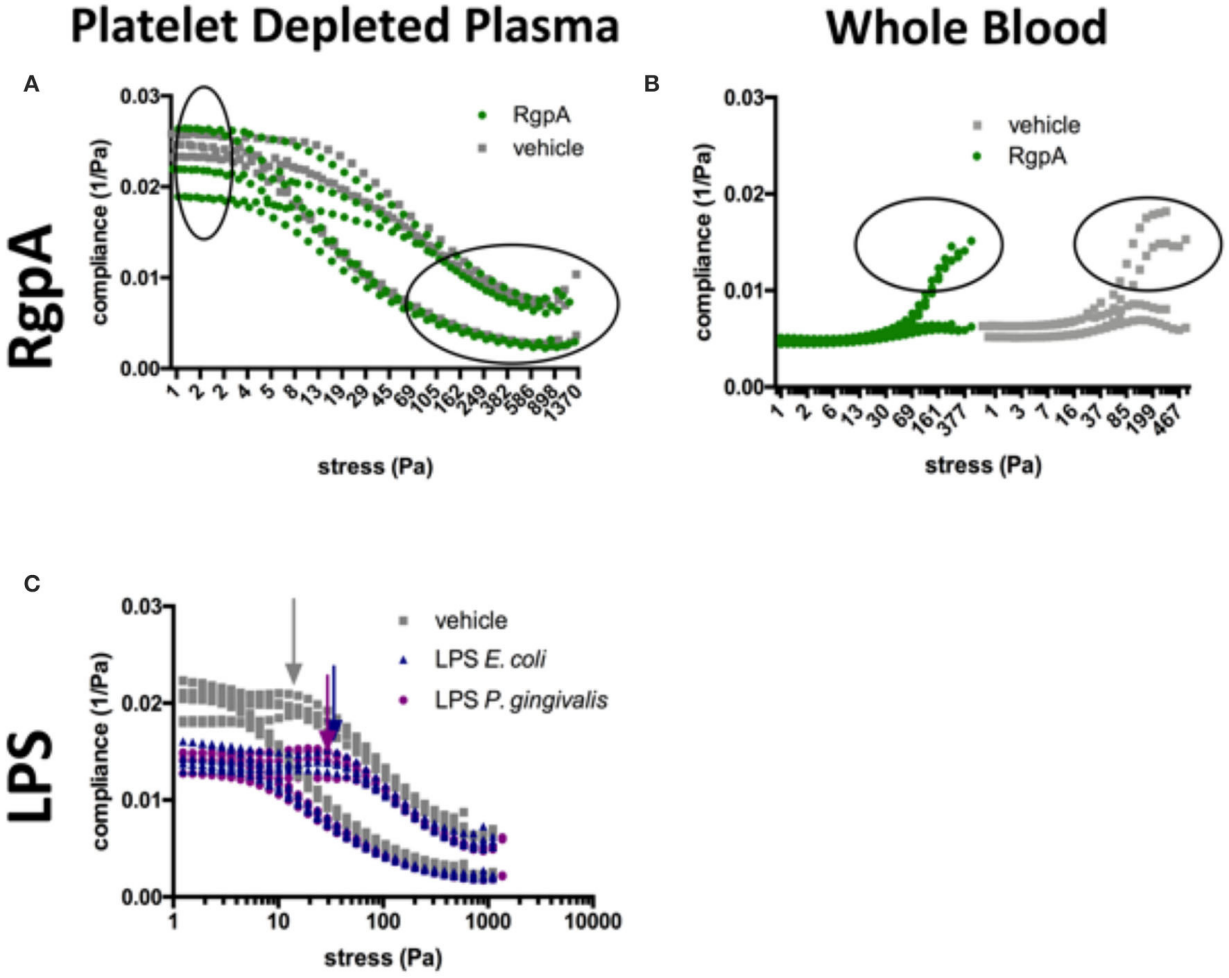
Compliance graphs of naïve vs. exposed samples, which reflect the intra-cycle behavior of clots during amplitude sweep tests. **(A)** RgpA-exposed PDP, **(B)** RgpA-exposed WB, **(C)** LPS-exposed PDP (*E. coli* and *P. gingivalis*). Graphs plot the sample runs in triplicate (PDP) and duplicate (WB).

Rheometry results for exposed samples and their matched control runs are recorded in Table 5 and Figure 7, that shows minimum (J'_M_) and large (J'_L_) strain compliance graphs of naïve vs. exposed samples, which reflect the behavior of clots at cyclic stress loading of clots during amplitude sweep tests. In PDP, all the different treatments increased the median linear elastic shear modulus of the clot, and led the non-linear response to start at similar and lower stress when compared to matched controls. However, the overlapping confidence intervals suggest a very small influence of the exposures on the G' modulus. Only exposure with LPS from *P. gingivalis* could increase the breakup stress.

**Table 5:** Rheometry data.

**Sample**	**G'_LVE_ [Pa]**	**Elastic limit [Pa]**	**Breakup stress [Pa]**
**Whole blood (WB)**			
Control 1	174.1 [156.2–192.0]	4.4 [4.4–4.4]	442 [305–578]
Control 1 + RgpA (100 ng·L^−1^)	201.9 [118.7–222.2]	4.5 [1.9–5.5]	341 [247–467]
Control 2	324.0 [291.5–332.1]	1.2 [1.2–1.2]	1,377 [1,372–1,705]
Control 2 + PG LPS (20 ng·L^−1^)	297.3 [261.7–314.1]	1.2 [1.2–1.5]	1,380 [1,376–1,702]
Control 2 + PG LPS (20 μg·L^−1^)	265.6 [211.8–284.6]	1.2 [1.2–1.9]	1,376 [1,374–1,377]
**Platelet depleted plasma (PDP)**			
Control 1	40.36 [38.58–42.71]	2.9 [2.9–2.9]	1,113 [1,109–1,370]
Control 1 + RgpA (250 ng·L^−1^)	45.52 [37.75–52.75]	2.3 [2.3–2.9]	1,110 [896–1,112]
Control 3	47.26 [44.56–54.78]	2.9 [1.9–5.4]	1,112 [584–1,113]
Control 3 + EC LPS (20 ng·L^−1^)	72.30 [61.77–76.84]	2.9 [2.9–3.6]	1,113 [898–1,116]
Control 3 + PG LPS (20 ng·L^−1^)	68.05 [67.60–77.92]	3.6 [2.9–3.6]	1,378 [1,112–1,379]

*Data presented as median [lower and upper confidence interval] of the triplicate runs. EC, E. coli; PG, P. gingivalis*.

Additional information can be gained from the compliances. LPS-exposure reduced both compliances (Figure 7C). They were also maintained over a larger shear stress range compared to their matched controls. Our model described in Figure 1 suggests that network alignment must be prolonged, which allows macroscopic shear-stiffening to start at higher stresses. The arrows in Figure 7C show this critical shear stress. Only after a shear stress of 380 Pa did the confidence intervals (CI) of the large-strain compliances (J'_L_) of LPS-clots and control clots overlap, indicating similar behavior of fully stretched clots after that. In RgpA-exposed PDP samples the compliances varied substantially at the start of the stress test (see also the high CI of the median G'_LVE_ value in Table 5), indicating that different network architectures have formed. During the stress tests, the compliances of the exposed and non-exposed samples converge completely (Figure 7A) indicating similar behavior of fully stretched clots.

In WB, the moduli were much higher than in PDP, but the RBCs blunted many effects that were seen in PDP, e.g., there was almost no shear-stiffening. Rather, WB clots showed a pronounced phase of shear-softening prior to the onset of weak shear-stiffening. In other words, the compliances increased until higher shear stress before they dropped. Other microscopic and macroscopic processes will take place in a stressed clot when blood cells are present. WB clot exposure to both high and low concentrations of *P. gingivalis* LPS resulted in a decrease of clot stiffness (which was more pronounced with the higher concentration), however, breakup stress was unaffected in both exposures. RgpA-exposed WB samples appeared to be stiffer when in near-equilibrium condition (see the median G'_LVE_ and also its high CI similar to PDP in Table 5) compared to their matched controls but they broke earlier while they were still in the phase of softening. It appears that RgpA exposure prevented shear-stiffening (Figure 7B).

## Discussion

Bacterial inflammagens in circulation can influence coagulation parameters that may result in abnormal clot formation and structure. Here we present the effects of gingipain R1 and LPS from *P. gingivalis* and *E. coli* on clot morphology and mechanics of clots produced from PPP, PDP, WB, as well as purified fibrinogen models. We point out that Jain et al. (56) reported that some LPS preparations might have lipoprotein contaminants present. Our LPS results on blood clots may therefore be due to LPS and/or the associated lipoprotein that might be present in the purchased LPS form Sigma. First looking at the morphology of LPS-exposed samples, SEM analysis in all our clot models showed that LPS from both *P. gingivalis* and *E. coli* caused the clot to become more dense and confluent in nature (Figures 2, 3). Individual, elongated fibers are visible in the controls, but in the exposures, the fibers are arranged to become dense and netlike (see Figure 2). This is in line with our previous analysis where LPS from *E. coli* caused fibers to become more netlike (in both PPP and fibrinogen models) (2). Our confocal and airyscan analyses on fluorescent fibrinogen (Figure 5) support the SEM observations. We also investigated the development of anomalous protein structures in PPP using autofluorescent signal when the LPS from *P. gingivalis* was added. Here, a significantly increased autofluorescent signal was assessed by the area analysis (Figure 4). These differences in fluorescent signal might reflect a structural change in the protein packaging in the presence of thrombin (61). Correlative AFM and Raman images from controls and *P. gingivalis* LPS-exposed samples (Figure 6), shows slight differences as band broadening, band position shift, and intensity ratio changes. Although the differences are small, they indicate a change in the local symmetry of the fibrinogen molecule in the C-H areas, localized on the fibers in the LPS-exposed samples (Figure 6E). This supports the altered morphological appearance of the clots as seen with SEM. This is the first report that shows LPS from *P. gingivalis* may chemically modify the structure of the fibrin(ogen) clot. These modifications may possibly be related to molecular changes of the fibrin(ogen) protein itself. Previously, we reported that LPS from *E. coli* added to fibrin(ogen) and plasma, can undergo structural changes that might be amyloid in nature. This was confirmed using the fluorescent amyloid stains, thioflavin T, and Amytrackers (52).

When RgpA is added to our different models, clots (viewed with SEM) were not confluent, but rather appeared mostly in sporadic clumps with masses of higher density surrounded by less dense areas. The fibers that did form showed a sparse and heterogeneous structure (see Figure 3). This was also previously established with confocal microscopy, where we added RgpA to fluorescent fibrin(ogen) and noted a decrease in formation of fibrin(ogen) networks (54). We propose that such heterogeneous clots will not be able to transmit hydrodynamic forces applied to them uniformly through the entire formed clot, but rather along their most elastic structures, while other parts of the clot remain mostly unstressed. This could pose excessive stress to existing structures, which could be a risk factor for clot pathologies. Our mechanical stress tests support this hypothesis (Figure 7B).

We further studied clot forming kinetics by 6 different parameters obtained from our TEG® tests and the mechanical response of our various clot models, before and after addition of LPS from *E. coli* and *P. gingivalis* as well as RgpA, by using rheometry. See Tables 1, 2 for the various parameters of TEG® and rheometry. After generating the WB and PDP clots in the cone-plate geometry of the rheometer we submitted them to increasing sinusoidal shear stresses and probed their strain responses. We also applied a model to differentiate the phase of network orientation at intermediate shear stresses from the subsequent phase of whole network stretch at higher shear forces.

RgpA-exposed clots seem to break abruptly at lower stresses in our WB model, whereas naïve clots showed a gradual yielding until the clot breaks at higher shear stresses (compare the encircled regions in Figure 7B). The same trend is seen in our PDP model, however, in this model breakup is not as abrupt as in the WB model. The heterogeneous structure seen in Figure 5 is also reflected by the high confidence interval of the linear elastic shear modulus and the compliances near equilibrium, however, the ability to shear-stiffen is unaffected (see Figure 7A). Our TEG® results showed that RgpA causes the PPP clot to form significantly slower (indicated by the *R*-value), and with a general alteration in clot strength (indicated by the MA and the TTG value). These results are consistent with the expectation that RgpA is a proteolytic enzyme. This is consistent with previous papers that looked at the proteolytic actions of RgpA on fibrinogen structure (48, 62). Our TEG® results also suggest that in the presence of LPS from *P. gingivalis*, the clot forms faster (R and TMRTG parameters), but the clot stiffness is not affected (MA and TTG). This is consistent with our previous results using LPS from *E. coli* (2, 52). These results are consistent with the finding of the rheometry by looking at the clot stiffness at its equilibrium (G'_LVE_). When we analyse our rheometry results further, in the PDP samples where we added the two LPSs the compliances were not only lower until 380 Pa applied shear stress, but shear-stiffening started also at higher stresses. This suggests that LPS-exposed clots will need more shear stress to stretch out all inhomogeneities before they can stretch like the control sample can (see Figure 7C, the arrows indicate this drop in the compliance, showing the shear stress where shear-stiffening begins). Such inhomogeneities are seen in our SEM samples as a denser and less uniform clot structure. Shear stiffening is a common property of biological fibers (63) and is *per se* not affected by LPS in our models. However, it is obvious that processes that soften the clot, such as fiber buckling and bending and network alignment, compete with processes that stretch the network and therefore shift the onset of macroscopic shear-stiffening to higher stresses.

## Conclusions

In this paper we bring together evidence that bacterial LPSs and RgpA can affect both clot structure and mechanics. This has significant implications for clotting and clot formation when these inflammagens enter into circulation, via various routes. These routes may include the gut when dysbiosis is present (leaky gut), the urinary tract (during infections), as well as the mouth area, during gingivitis and periodontitis. It is well-known that these entry pathways are active in most inflammatory conditions. When in circulation, these inflammagens interacts with soluble fibrinogen, where they bring about all the effects we have described (mechanical and structural changes). In future, it would also be valuable to investigate the effects of Kgp, and RgpB, and combinations of the molecules with RgpA, as all of these molecules contribute significantly to the virulence of the bacterium (64, 65). Ultimately, these interactions are associated with systemic inflammation and coagulation pathologies. The magnitude of this effect differs in plasma and purified fibrinogen and most likely exists due to the presence of inhibitory and target molecules in plasma such as albumin and other proteins. The presence of these inflammagens in the circulation of individuals with various cardiovascular and systemic inflammation conditions, including T2DM, may have far-reaching healthy effects on blood clotting.

## Data Availability Statement

The raw data supporting the conclusions of this article will be made available by the authors, without undue reservation. The datasets generated as well as figure micrographs analyzed during the current study are available: https://1drv.ms/u/s!AgoCOmY3bkKHioRESgGKZsHuntFsoA?e=BTUXvr.

## Ethics Statement

The studies involving human participants were reviewed and approved by Stellenbosch University Human Ethics Committee. The patients/participants provided their written informed consent to participate in this study. Ethics was also obtained from the Ethics Committee of the Medical University Vienna, Austria.

## Author Contributions

JN: TEG®, rheometry, and SEM. TF: TEG® and SEM. MP: rheometry, SEM, confocal, and data analysis. CV: technical assistance. UW: rheometry. EP: study leader. All authors approved submission of the paper. DK: editing of paper and co-corresponding author. OL: raman analysis.

## Conflict of Interest

OL is employed by the company, Horiba. She analysed the sample using Raman technology. The remaining authors declare that the research was conducted in the absence of any commercial or financial relationships that could be construed as a potential conflict of interest.

## Abbreviations

LPS: lipopolysaccharides
LTA: lipoteichoic acids
IDDM: Dysregulation and Dormant Microbes
AD: Alzheimer's disease
PD: Parkinson's disease
T2DM: Type diabetes
P. gingivalis: Porphyromonas gingivalis
TLR4: Toll-like Receptor 4
Rgp: Arg-gingipain
Kgp: Lys-gingipain
Rgp(A) and (B): arginine gingipains and recombinant *P. gingivalis* gingipain R1 (RgpA)
ILs: Interleukins (ILs)
E. coli: Escherichia coli
PPP: Platelet poor plasma
PDP: platelet-depleted plasma
HMDS: hexamethyldisilazane
OsO_4_: osmium tetroxide
TEG®: Thrombelastograph®
LVE: linear viscoelastic behavior
J'_M_: minimum-strain compliance
J'_L_: large-strain compliance.
